# A rare case of extra-digital glomus tumor of the thigh

**DOI:** 10.1016/j.ijscr.2024.110246

**Published:** 2024-09-06

**Authors:** Camil Chouairy, Souad Ghattas, Hani Maalouf, Ahmad Youness, Jad El Bitar, Mansour El Khoury

**Affiliations:** a Chairman of Anatomic Pathology Department, Saint Georges Hospital University Medical Center, Beirut, Lebanon; b Department of General Surgery, Saint Georges Hospital University Medical Center, University of Balamand, Beirut, Lebanon; c Saint Georges Hospital University Medical Center, University of Balamand, Beirut, Lebanon

**Keywords:** Glomus tumor, Extra-digital glomus tumor, Thigh

## Abstract

**Introduction:**

Glomus tumors are rare benign tumors arising from glomus bodies that are responsible for thermoregulatory control. Their typical location is the subungual area of the digits, and extra-digital glomus tumors are very rare, leading to misdiagnosis and delayed treatment due to the absence of typical symptoms.

**Case:**

Here, we report the case of a 49 years old male patient with a long history of localized right thigh pain who was found to have an extra-digital glomus tumor of the thigh after surgical excision.

**Discussion:**

A comprehensive physical examination, detailed medical history, in depth imaging and early surgical excision upon clinical suspicion may prevent delayed or incorrect diagnosis. The treatment of glomus tumor is surgical excision providing immediate relief from pain, however if the lesion is not palpable, it can be easily missed or confusing with other diagnoses such as schwannoma, neuroma or arteriovenous malformation.

**Conclusion:**

Glomus tumors of the thigh represent an exceptional location for extra digital glomus tumors. The aim of this report was to make the surgical community more aware of this entity to prevent delayed treatment and misdiagnosis. Glomus tumor should be kept in mind in the differential diagnosis of all painful subcutaneous lesions.

## Highlights

• Glomus tumors are benign neoplasms containing cells from the glomus apparatus which is responsible for thermoregulatory control. It is a rare tumors accounting for only 1-2% of all soft tissue tumors that usually presents as a painful subcutaneous nodule formation located on the subungual area of the digits.
• Extra-digital glomus tumors are a very rare presentation and the location in the thigh is exceptional with only a few cases reported in the literature.
• The treatment of glomus tumor is surgical excision providing immediate relief from pain, however if the lesion is not palpable, it can be easily missed or confusing with other diagnoses such as schwannoma, neuroma or arteriovenous malformation.
• Glomus tumor should be kept in mind in the differential diagnosis of all painful subcutaneous lesions.

## 1 Introduction

Glomus tumors are benign neoplasms containing cells from the glomus apparatus which is responsible for thermoregulatory control [1]. It is a rare tumors accounting for only 1–2 % of all soft tissue tumors that usually presents as a painful subcutaneous nodule formation located on the subungual area of the digits [2]. Extra-digital glomus tumor's locations are even rarer leading to misdiagnosis and delayed treatment due to the physician's lack of awareness and low level of suspicion [3]. The work has been reported in line with the SCARE criteria [4].

We report here the case of a 49 years old male patient with a long history of localized right thigh pain found to have an extra-digital glomus tumor of the thigh after surgical excision.

## 2 Case

A 49-year-old male patient presented with localized pain in the medial aspect of the right thigh. The pain started insidiously 5 years ago, exacerbated by movement or upon palpation and not relieved by anti-inflammatory. As past medical history patient is known to have dyslipidemia and negative past surgical history. On Physical exam there were any palpable mass on the right thigh. The overlying skin was normal without any evidence of inflammation. Range of motion and neurological examination of the right lower limb were normal. Patient denied any traumatic injury to the thigh.

The patient saw multiple physicians and multiple imaging including ultrasound and Magnetic resonance imaging (MRI) were done without final diagnosis. MRI done two years ago showed an enhancing 5 mm nodule in the subcutaneous fat of the medial distal thigh showing a well-defined border and without infiltration of the adjacent muscles. The subcutaneous location and the stable size since prior examination were suggestive of benign etiology.

**Fig. 1:**
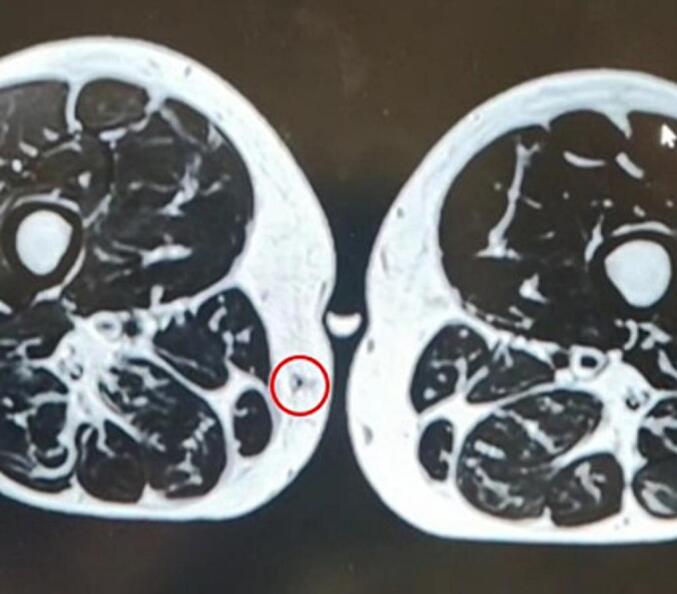
Thigh MRI with gadolinium, T1 sequence, showing a 4 × 2 mm hypointense, oval-shaped nodule (Red circle) located in the subcutaneous area far from the dermis on the median right thigh. (For interpretation of the references to colour in this figure legend, the reader is referred to the web version of this article.)

When patient presented to our care, repeat imaging and laboratory studies were done. Biology, including Complete blood count, C-reactive protein and erythrocyte sedimentation rate were within normal limits. Echo Doppler showed at the level of the painful area on the right medial thigh, a non-specific non-inflammatory granulomatous mass of 4 × 2mm, not vascularized and 5 mm deep from the skin. Thigh MRI with gadolinium showed a 4 × 2 mm oval-shaped nodule located in the subcutaneous area far from the dermis on the median right thigh and shows 2 tiny vessels artery and vein traversing it. Findings were suspicious of angioma or arteriovenous malformation, but schwannoma or neuroma can't be ruled out (Fig. 1).

**Fig. 2:**
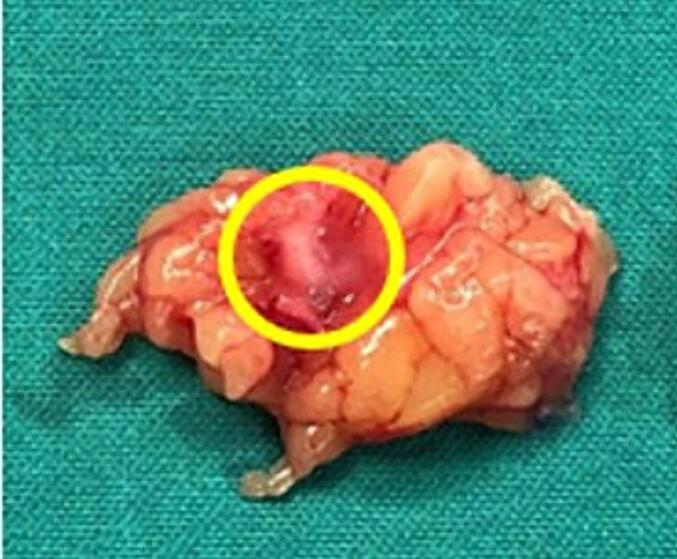
Specimen removed consistent of fat tissue+ glomus tumor measuring 8 mm that was firm and differentiated from the fat by its tissue density (Yellow Circle). (For interpretation of the references to colour in this figure legend, the reader is referred to the web version of this article.)

**Fig. 3:**
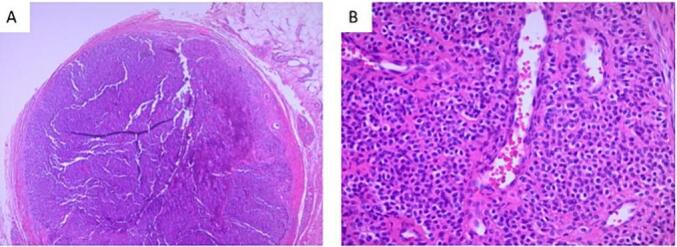
a-Well circumscribed encapsulated Nodule (H&E) (25× Original Magnification). b-Sheets of uniform cells with round nuclei interrupted by vessels of variable size (H&E) (200× Original Magnification).

**Fig. 4:**
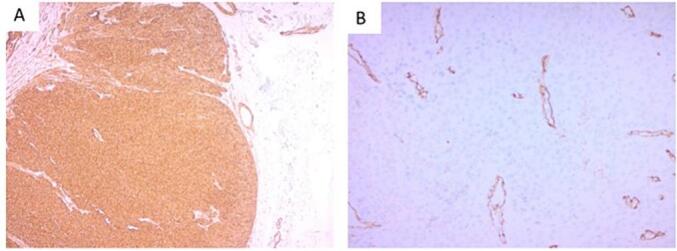
a-Smooth Muscle Actin (SMA) Immunohistochemical stain, showing strong expression by the neoplastic cells (50× Original Magnification). b-CD34 Immunohistochemical stain highlighting the endothelial cells of the vessels surrounded by tumor cells (200× Original Magnification).

Facing diagnosis difficulties, an excision of the mass was performed. The operation was done under local anesthesia with lidocaine injection. Extensive dissection was performed following the site of the nodule seen on MRI till reaching the mass that was excised en bloc with negative macroscopic margins. The mass was firm and differentiated from the fat by its tissue density (Fig. 2). Pathological examination of the specimen revealed mostly normal mature adipose, in the center of which there was an 8 mm solid well circumscribed encapsulated nodule. The nodule was composed of sheets of uniform cells, interrupted by vessels of variable size. The neoplastic cells exhibited round nuclei with regular contour and well-defined outlines (Fig. 3). Necrosis and mitotic figures were absent. The neoplastic cells showed strong expression of Smooth Muscle Actin (SMA). EMA and S-100 stains were Negative. CD34 immunohistochemical stain highlighted the endothelial cells of the vessels that were surrounded by the neoplastic cells (Fig. 4).

Postoperatively, the patient was immediately relieved from pain and discharged home on the same day. Follow up at 2 months post operatively, the patient was pain free.

## 3 Discussion

Glomus bodies, a neuro-myo-arterial structure located at the dermo-hypodermic junction, are the source of glomus tumors, which are uncommon benign tumors. The glomus bodies' function is to control blood flow to the skin in response to variations in body temperature. Less than 1 % of all soft tissue cancers are globus tumors [2]. These can be categorized as extra-digital or digital/subungual, solitary or numerous, sporadic or familial lesions [5]. Although glomus bodies are present all over the body, they are most heavily concentrated in the hands, feet, and palms, which explains why the subungual region and lateral portions of the fingers are where they are most frequently found [6]. There are extremely few examples of extra-digital glomus tumors documented in the literature, and the location in the thigh is unusual [7]. Only five out of the 56 individuals with extra-digital glomus tumors had the tumor located in their thighs [7]. Because of the deep placement, tiny tumor size, and lack of particular symptoms, it is frequently misdiagnosed and treated incorrectly [6].

Hand glomus tumors typically exhibit the traditional symptoms of pain and cold intolerance. The lack of normal symptoms makes extra-digital glomus tumor diagnosis more challenging. In their investigation of five individuals with extra-digital glomus tumors, Temiz et al. noted that a consistent finding was a purple subcutaneous lump that felt quite painful when palpated [6]. Lee et al. compared 42 individuals with extra-digital glomus tumors to 110 patients with digital glomus tumors and discovered that single purplish papules were the most typical clinical manifestation of extra-digital glomus tumors. When it came to extra-digital glomus tumors, the male to female ratio was 4:1, however in digital glomus tumors, the female predominance was 2:1 [7]. Our patient was suffering from pain that is exacerbated by movement and tenderness to palpation without hypersensitivity to cold. And the mass had no skin discoloration and wasn't palpable on physical exam so it was difficult to suspect a glomus tumor at this stage.

It has been proposed that MRI and ultrasound imaging can help with glomus tumor diagnosis. A generic, well-demarcated, rounded, hypoechoic mass that is hypervascularized on doppler is shown on ultrasound imaging. MRI especially in high resolution represents the most sensible and specific tool distinguishing between the tumor itself and the normal tissue. When gadolinium is injected, magnetic resonance imaging reveals a low signal intensity on T1 weighted pictures and a high signal intensity on T2 weighted images, particularly around the edge. After histological analysis, complete excisional biopsy is still the most effective method for reaching a definitive diagnosis [3].

A few clinical characteristics, such as big size, deep soft tissue or visceral location, infiltrative growth pattern, or multicentricity, might arouse the suspicion of a malignant glomus tumor, albeit the conversion to a malignant tumor is extremely rare—less than 1 % of instances have been recorded to occur [8,9].

The treatment of glomus tumor is surgical excision providing immediate relief from pain, however if the lesion is not palpable, it can be easily missed or confusing with other diagnoses such as schwannoma, neuroma or arteriovenous malformation [[1], [2], [3], [4], [5], [6], [7], [8], [9]]. Small lesions can be treated with laser ablation using argon or carbon dioxide lasers, sclerotherapy using sodium tetradecyl or hypertonic saline solution [[4], [5], [6], [7], [8], [9]]. Recurrence after surgical resection is infrequent rates around 12 % in different publications and could be related to inadequate surgical excision or multiple primary tumors [[3], [4], [5], [6], [7], [8], [9], [10], [11]].

## 4 Conclusion

Glomus tumors of the thigh represent an exceptional location of extra-digital glomus tumors. They usually present with pain and localized tenderness. The diagnosis is frequently delayed due to lack of awareness among physicians and low level of suspicion. A comprehensive physical examination, detailed medical history, in depth imaging and early surgical excision upon clinical suspicion may prevent delayed or incorrect diagnosis. Glomus tumor should be kept in mind in the differential diagnosis of all painful subcutaneous lesions.

## Informed consent

Written informed consent was obtained from the patient for publication and any accompanying images. A copy of the written consent is available for review by the Editor-in-Chief of this journal on request.

## Ethical approval

Case report approved for publishing by ethical committee at Saint George hospital University Medical Center.

## Funding

None.

## Author contribution

**Camil Chouairy MD**: supervision, validation, project administration, conception of the work, revising the work critically for important intellectual content and final approval of the version to be published project administration, **Souad Ghattas MD**: first Author, conception of the work, design of the work, review of literature, draft manuscript, revising the work critically for important intellectual content, final approval of the version to be published, **Hani Maalouf MD**: review of literature, draft manuscript, revising the work critically for important electual content, final approval of the version to be published, **Ahmad Younes MD**: conception of the work, design of the work,review of literature, draft manuscript, revising the work critically for important electual content, final approval of version to be published, **Jad El Bitar MD**: review of literature, draft manuscript, **Mansour El Khoury MD**: supervision, validation, project administration, conception of the work, revising the work critically for important intellectual content, final approval of the version to be published project administration and corresponding Author.

## Guarantor

Souad Ghattas.

## Research registration number

N/A.

## Conflict of interest statement

The authors report no conflict of interest.

## Data Availability

Consent to publish was obtained from the patient for publication of this case report and accompanying images. All Data are available for review by the Editor-in-Chief of this journal on request.
